# Surgery

**Published:** 1879-03

**Authors:** 


### SURGERY.

VOLKMANN AND KRASKE. Report of the surgical clinic at Halle. Sixth annual meeting of the German Society of Surgery. (*Contralbl. f. Ch.* No. 50, 1878.)

The report contains only the larger amputations (including those of the breast), resections, operations for hydrocele, complicated fractures, and penetrating wounds of joints. (It must be remarked, that at no other clinic are the antiseptic principles more strictly obeyed than at Halle.)

There were, altogether, 183 exarticulations and amputations performed on 172 patients (*not* counting amputations of fingers, toes, and partial amputations of the hand).

Of these, 139 cases were uncomplicated, with a mortality of 2.87 per cent. (4 cases). One case of traumatic exarticulation of the humerus, and one of the thigh (also traumatic), died four hours after the operation. An amputation of the leg was followed by death from habitual erysipelas, while only one case of amputation of the thigh (out of 42) in which the injury extended beyond the nates, died after twenty-four hours.

Among the complicated cases there were:

(1.) Nine double amputations, with two cases of death from shock (amputation of the thighs).

(2.) Six amputations, complicated with severe multiple injuries, all fatal.

(3.) Fifteen amputations, performed on patients suffering from septicæmia or pyæmia, before entering the hospital; of these 8 died (52.3 per cent.)

(4.) Three patients, who died from intercurrent diseases, not depending on the surgical affection, viz: One delirium tremens, 1 genuine pneumonia, 1 case of abortion, followed by puerperal fever.

Therefore, of 172 patients, with 183 amputations, there died 23 cases (with 27 amputations), while 149 (with 156 amputations) recovered.

Ninety-one uncomplicated resections of large joints (48 hip-joints) were performed, with a mortality of 5 cases. Two children of three-fourths and 2½ years, died in collapse; 1 patient succumbed to thrombosis of both crural veins, another to hæmorrhage from involvement of the crural artery in a lymph-gland abscess. One child died of basilar meningitis three weeks after the operation.

Four re-sections, complicated by previously existing hernia, proved fatal. In 2 other cases fatal complications existed in the shape of acute phthisis and of delirium tremens.

No cases were lost among 10 re-sections in the continuity of the bone. Among 50 osteotomies (necrosis operation) 1 case of hæmorrhage in hæmophilia represented the total mortality.

One hundred and nineteen amputations of the mammary gland were performed on 110 patients; the contents of the entire axilliary cavity were included in the operation seventy-five times. Six patients died, viz.: two of erysipelas, one in collapse a few hours after the operation, one from malignant pustule (infected with catgut), and two of marasmus from chronic suppuration.

Forty-five cases of hydrocele were treated by incision without any mortality. No deaths occurred, likewise, amongst *seventy-three cases of complicated fracture and twenty-four instances of penetrating wounds of joints.*

Pyæmia did not occur in a single case, septicæmia only in three cases of gangrene of the leg after ligation of the crural artery. Chronic septicæmia was observed very rarely; tetanus and erysipelas were each observed four times.

(Hitherto the partisans of Lister have referred to the success and shortened duration of single cases rather than to statistics, in order to prove the efficacy of the antiseptic method. With such statistics, however, as those of Volkmann, they can enter boldly into the ranks.)

